# Effect of Metakaolin on Strength and Efflorescence Quantity of Cement-Based Composites

**DOI:** 10.1155/2013/606524

**Published:** 2013-04-28

**Authors:** Tsai-Lung Weng, Wei-Ting Lin, An Cheng

**Affiliations:** ^1^Physics Division, Tatung University, 40 Zhongshan North Road, 3rd Section, Taipei 104, Taiwan; ^2^Deptartment of Civil Engineering, National Ilan University, 1 Shen-Lung Road, Ilan 260, Taiwan; ^3^Institute of Nuclear Energy Research, Atomic Energy Council, Executive Yuan, Taoyuan 325, Taiwan

## Abstract

This study investigated the basic mechanical and microscopic properties of cement produced with metakaolin and quantified the production of residual white efflorescence. Cement mortar was produced at various replacement ratios of metakaolin (0, 5, 10, 15, 20, and 25% by weight of cement) and exposed to various environments. Compressive strength and efflorescence quantify (using Matrix Laboratory image analysis and the curettage method), scanning electron microscopy, and X-ray diffraction analysis were reported in this study. Specimens with metakaolin as a replacement for Portland cement present higher compressive strength and greater resistance to efflorescence; however, the addition of more than 20% metakaolin has a detrimental effect on strength and efflorescence. This may be explained by the microstructure and hydration products. The quantity of efflorescence determined using MATLAB image analysis is close to the result obtained using the curettage method. The results demonstrate the best effectiveness of replacing Portland cement with metakaolin at a 15% replacement ratio by weight.

## 1. Introduction

Efflorescence is a fine, white, powdery deposit of water-soluble salts left on the surface of concrete as the water evaporates. This deposit is detrimental to the durability of cementitious materials and a stubborn problem for researchers in the field of masonry and concrete [1]. Until recently, it was assumed that calcium hydroxide (Ca(OH)_2_, CH) forming within cement-based composites is responsible for efflorescence; however, CH does not contribute sufficiently towards the soluble alkali sulfates required for efflorescence to occur. Alkali sulfates penetrate through pores within the composites toward the surface. Reducing the number and size of these pores restricts the movement of salts to the surface. One approach is consolidating grout through mechanical vibration to reduce voids in the grout while improving the bond between the steel and the masonry wall. Producing composites with a denser microstructure also reduces the porous nature of the material, making it difficult for salts to migrate [2, 3].

In recent years, supplementary cementitious materials (SCMs), such as fly ash, slag, and silica fume, have been used to replace a portion of the aggregate or cementitious material in cement-based composites. The aim has been to improve the mechanical properties by taking advantage of their extremely fine spherical particles [4–7]. The pozzolanic reaction of SCMs produces an additional binder, which increases the density of the microstructure, thereby reducing permeability. The problem of efflorescence can be greatly reduced by including SCMs in cement-based composites.

Metakaolin has been widely studied for its highly pozzolanic properties, suggesting that metakaolin could be used as an SCM. Unlike other SCMs that are secondary products or by-products, metakaolin is a primary product, obtained by calcining kaolin clay within a temperature range of 650 to 800°C [8, 9]. Metakaolin is increasingly being used to produce materials with higher strength, denser microstructure, lower porosity, higher resistance to ions, and improved durability [10–12]. 

Very few researchers have addressed the problem of efflorescence in metakaolin cement-based composites. This study sought to determine the appropriate quantity of metakaolin required (as a replacement for cement) to reduce efflorescence. We employed specimens with various replacement ratios of metakaolin (0%, 5%, 10%, 15%, 20%, and 25%) at a water/cement (w/c) ratio of 0.5. The occurrence of white efflorescence was investigated under various curing environments, at the curing age of 3, 7, and 28 days. 

## 2. Experimental Program

### 2.1. Materials and Specimens

We produced matrices of ASTM Type *Ι* Portland cement, silica sand, tap water, and metakaolin. The specific gravity and fineness modulus of the silica sand were 2.64 and 2.40, respectively. The physical and chemical properties of the metakaolin are presented in Tables 1 and 2. 

Metakaolin was added as a replacement for cement at the following percentages: 0, 5, 10, 15, 20, and 25% of the weight of cement with the water/cementitious ratio (w/c) set to 0.50. The mixes were then exposed to the following environments: normal environment (NE), 25°C with 85% humidity; carbon dioxide environment (CDE), in a carbonization tub with 100% carbon dioxide at 15 atm pressure, 100°C, and 90% relative humidity; low temperature environment (LTE), refrigerated at −5~0°C with 2% humidity. 

The mix proportions are presented in Table 3. The coding in Table 3 (M0, M5, M10, M15, M20, and M25) represents the percentage of metakaolin. 

Cubic specimens (50 × 50 × 50 mm) were prepared to test the compressive strength. Additional specimens (150 × 150 × 30 mm) were also prepared for the quantification of efflorescence using image analysis in MATLAB. Finally, specimens (10 × 10 × 10 mm) were sliced from the mortar specimens for observation under scanning electron microscope (SEM) and samples of mortar powder (3 g) were prepared for X-ray diffraction (XRD).

### 2.2. Testing Methods

Compressive strength was determined after 1, 3, 7 and 28 days of curing, according to ASTM C109-12. The extent of white efflorescence was quantified according to RGB values using MATLAB (Matrix Laboratory) image analysis of photographs (taken at 7 and 56 days) of samples exposed to the three experimental environments (NE, CDE, and LTE). MATLAB image analysis was unable to determine the thickness of efflorescence; therefore, the specimens were analyzed using the curettage method to quantify efflorescence according to weight. The curettage method indicated that we removed the efflorescence with a spatula and then weighed it.

A petrographic examination of hardened mortar was performed using SEM according to ASTM C856-11 specifications. The specimens were dried, vacuumed, and Au ion-sputtered prior to SEM investigation in order to render the surface conductive. By varying the degree of magnification (×1000 and ×3000), capillary pores of various sizes (micro- or meso-pore structure) were estimated. 

We also investigated the compounds of cement-based materials following replacement with metakaolin. Because the hydration of composite materials is multiphased, all of the compositions are compounds; that is, almost no single element structures exist. As a result, we employed XRD analysis to analyze the chemical compounds within cement-based materials. These samples were ground into powder at room temperature under air-dried conditions and XRD patterns were recorded using Cu-K radiation between 20° to 80°, at a scanning speed of 0.5 s/1°. The composition of the compounds was determined by comparing XRD intensity diagrams with the peak values of corresponding compounds in the computer database. 

## 3. Results And Discussion

### 3.1. Compressive Strength

**Figure 1:**
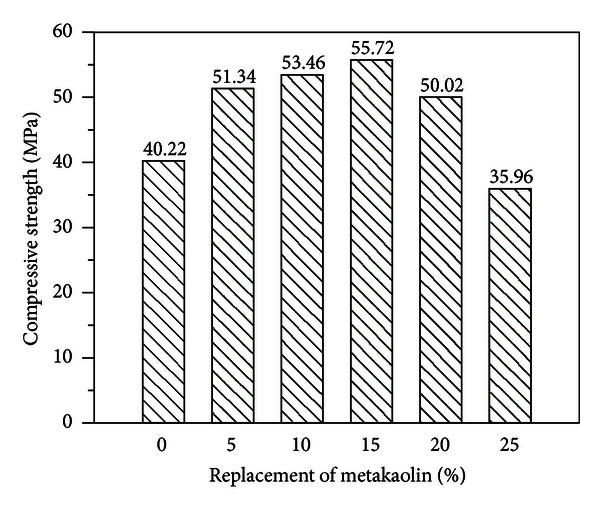
Histogram: compressive strength versus replacement of metakaolin (age = 28 days).

 Compressive strength and the percent of relative compressive strength are presented in Table 4. At the curing age of 28 days in the experiment results, the highest compressive strength was obtained from specimens with 15% metakaolin (as a replacement for cement), as illustrated in Figure 1. The compressive strength of specimens with metakaolin increased with time; however, for specimens containing 25% metakaolin, the strength characteristics failed to meet those of standard mortar specimens. The inclusion of metakaolin in cement-based composites enhances compressive strength through the filler effect in the interfacial transition zone between the cement paste and aggregate particles. In addition, CH gels are quickly removed during the hydration of cement with metakaolin and actually accelerate cementitious hydration. 

### 3.2. Quantification of Efflorescence Using MATLAB Image Analysis

The specimens were photographed at the curing age of 7 and 56 days to quantify efflorescence using MATLAB image analysis. The areas affected by efflorescence as a ratio of the total area are summarized in Table 5. The specimens containing 15% metakaolin present a distinctly lower degree of efflorescence, compared to the other specimens, according to the presence of deposits both on and around the specimens. 

**Figure 2:**
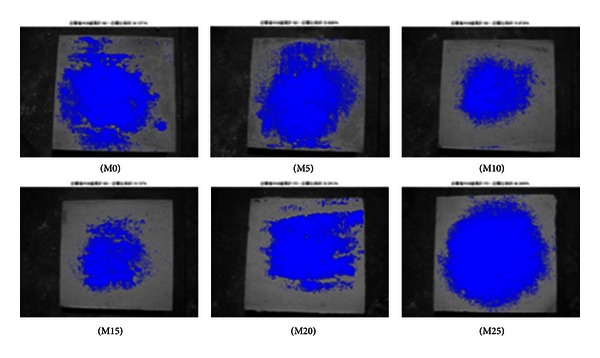
Quantity of efflorescence under normal environmental conditions (7 days).

**Figure 3:**
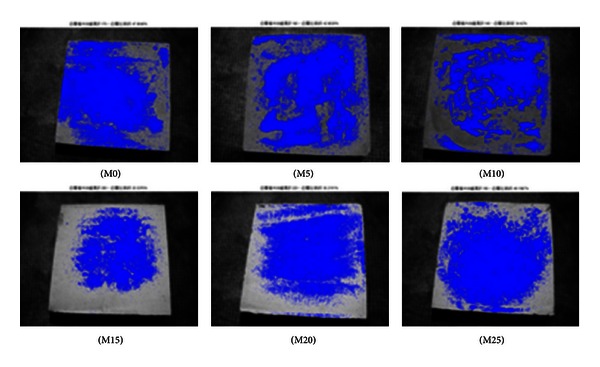
Quantity of efflorescence under normal environmental conditions (56 days).

**Figure 4:**
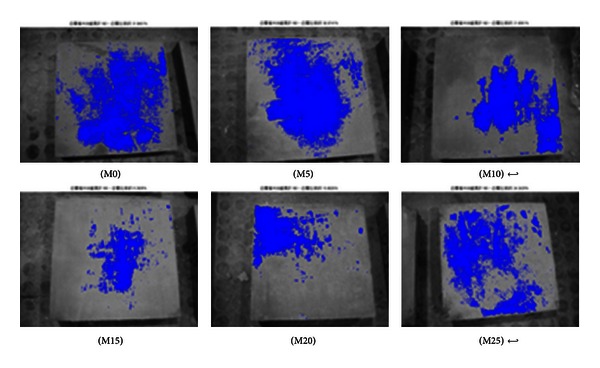
Quantity of efflorescence under carbon dioxide environmental conditions (7 days).

**Figure 5:**
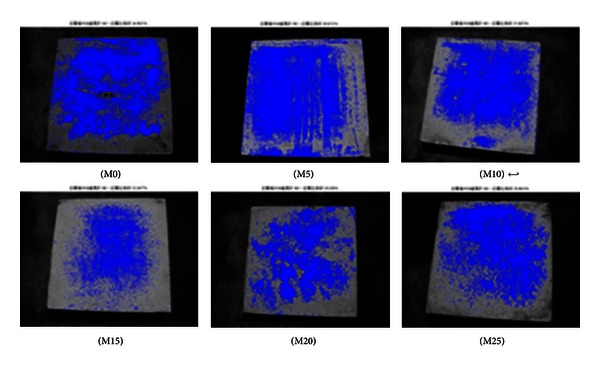
Quantity of efflorescence under carbon dioxide environmental conditions (56 days).

**Figure 6:**
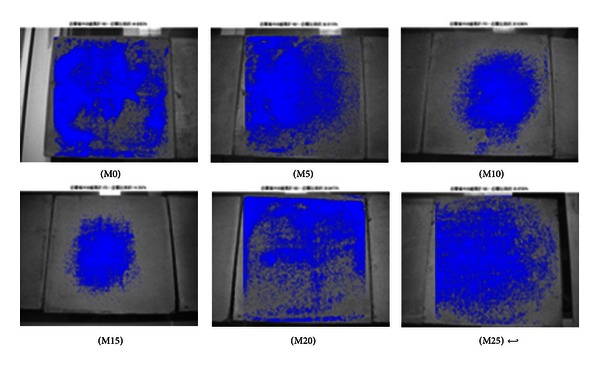
Quantity of efflorescence under low temperature environmental conditions (7 days).

**Figure 7:**
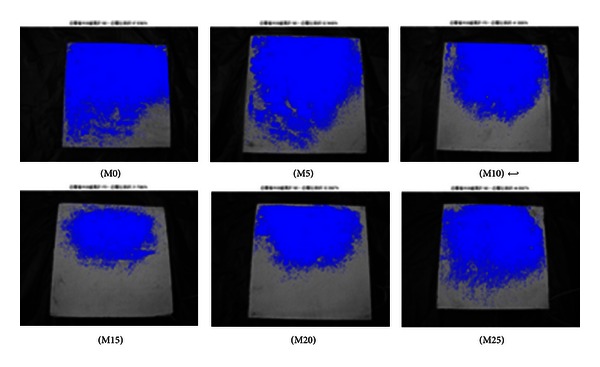
Quantity of efflorescence under low temperature environmental conditions (56 days).

**Figure 8:**
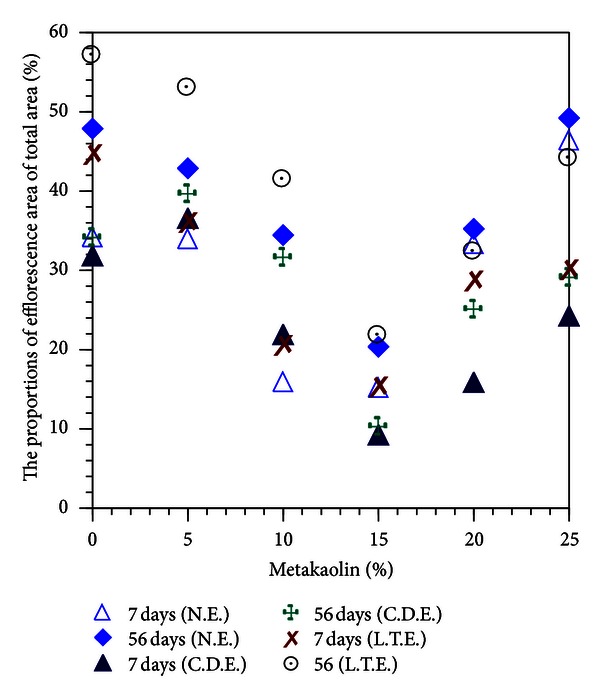
Proportional area of efflorescence versus replacement of metakaolin.

As shown in Figures 2, 3, 4, 5, 6, and 7, the most obvious efflorescence was observed on specimens cured under LTE conditions. In addition, the inclusion of metakaolin decreased the extent of efflorescence in all specimens except for those with 20% and 25% metakaolin; specimens with 15% metakaolin showed the least efflorescence. These results verify that the inclusion of metakaolin can accelerate the hydration reaction with CH to produce a denser, more homogeneous material with a narrower transition zone, thereby reducing the extent of efflorescence. These results are in strong agreement with those of the relationship between efflorescence area and the replacement of metakaolin, as shown in Figure 8. 

**Figure 9:**
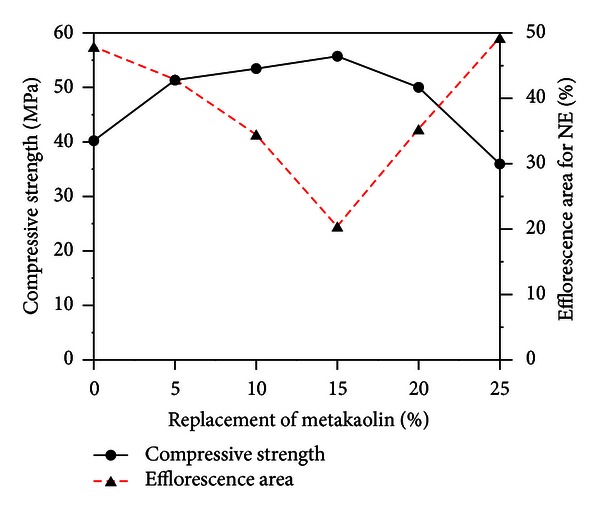
Curves comparing compressive strength versus replacement of metakaolin and area of efflorescence versus replacement of metakaolin (NE specimens).

Figure 9 compares the area affected by efflorescence with compressive strength values; the data are fundamentally consistent with the visible extents of efflorescence in Figures 2, 3, 4, 5, 6, and 7, for NE specimens. As shown in Figure 9, cement-based composites produced with higher quantities (5% to 20%) of metakaolin provide higher strength and resistance to efflorescence, due to a denser microstructure and the controlled concentration of mobile alkalis in the pore solutions, respectively. 

### 3.3. Quantification of Efflorescence Using the Curettage Method

The results for the quantification of efflorescence using the curettage method are presented in Tables 6, 7, and 8. Clearly, the addition of 15% metakaolin was most effective in inhibiting efflorescence under any exposure environment. The specimens cured under LTE conditions had a higher quantity of efflorescence than those under NE or CDE. Based on the previous study, higher humidity led to a higher quantity of efflorescence, and efflorescence increased with an increase in the size of moist particles [13]. In addition, efflorescence was proportional to alkali leaching, perhaps due to the larger volume of macropores (particularly those between 200 nm and 1000 nm) capable of increasing the diffusion coefficient for the migration of Na from geopolymer phase into the solution [14]. 

### 3.4. Effect of Metakaolin on Microscopy Characteristics

**Figure 10:**
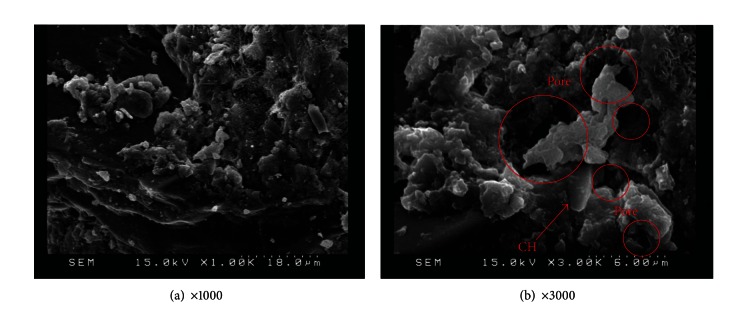
SEM observations for M0 specimens.

This study employed SEM observations to characterize the microstructural compounds produced with/without replacement metakaolin. Careful analysis of microstructures can reveal the pozzolanic reaction and the gel development. SEM magnification was set at 1,000 and 3,000 times to directly observe the development of cement hydration and pore structure. SEM observation was performed on control specimens after 56 days of aging, shown in Figure 10; large capillary pores, CH, and pore interconnectivity were observed. 

**Figure 11:**
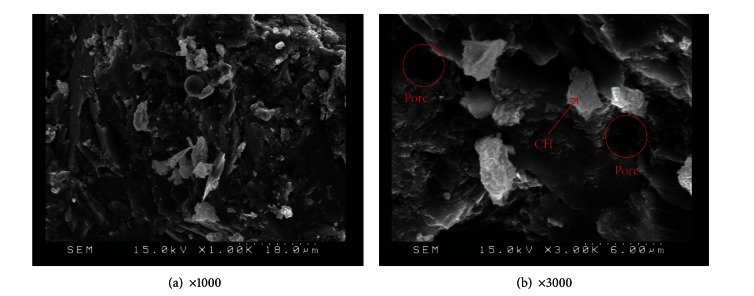
SEM images of M5 specimens.

**Figure 12:**
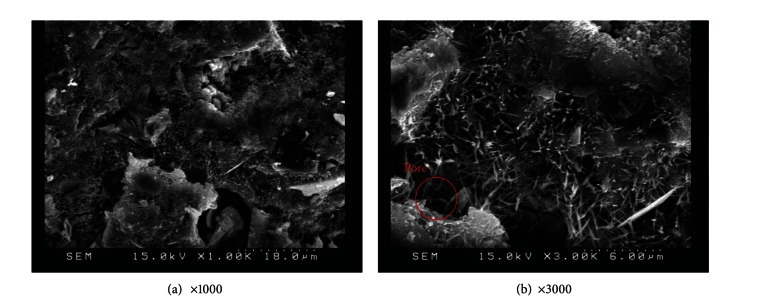
SEM images of M10 specimens.

**Figure 13:**
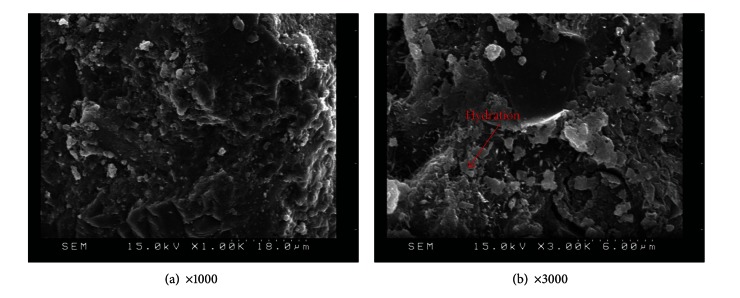
SEM images of M15 specimens.

**Figure 14:**
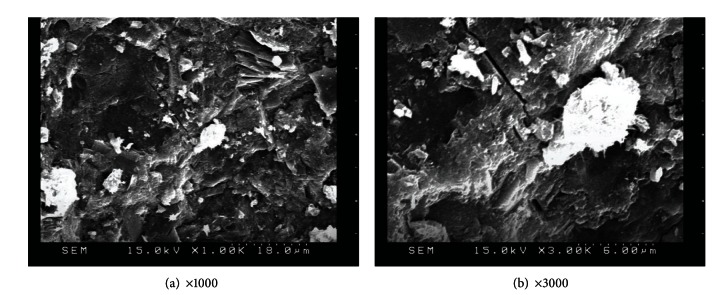
SEM images of M20 specimens.

**Figure 15:**
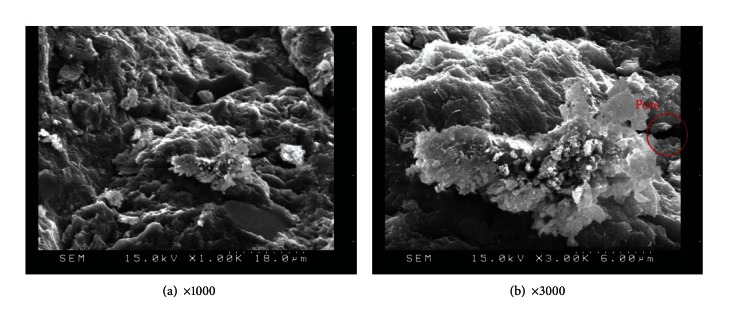
SEM images of M25 specimens.

SEM observation was also applied to specimens with various amounts of replacement metakaolin (5%, 10%, 15%, 20%, and 25%) at 56 days, as shown in Figures 11, 12, 13, 14, and 15. Clearly, cement-based paste specimens with replacement metakaolin developed a more compact, denser pore structure. Hydration was formed on the surface of the M5, M10, M15, and M20 specimens; the microstructure of these samples reduced the mobility of chloride and other ions, resulting in higher compressive strength and a reduction in crack stretching. 

The SEM image in Figure 13 illustrates the relatively dense, homogeneous microstructure of M15 specimens with smooth surfaces and no distinct pores. The dense microstructure observed in SEM images is consistent with the results of compressive strength. A number of large capillary pores were observed in specimens containing 25% metakaolin, as illustrated in Figure 15, exceeding those in other samples. This may be due to the fineness of metakaolin reducing the workability of the composites, resulting in inconsistent dispersion of particles throughout the specimens. In addition, the 25% metakaolin specimens contain a lot of unreacted precursor powder due to the lack of water.

A pozzolanic reaction between metakaolin and CH occurred during the hydration of cement, resulting in the consumption of a portion of the CH. The formation of secondary calcium-silicate-hydrate (C-S-H) gel (due to pozzolanic reaction), although less dense than the primary C-S-H gel, is effective in filling and segmenting large capillary pores into small, discontinuous capillary pores through pore size refinement, thereby decreasing the total permeability of cement-based composites. The filler action of metakaolin due to its fine particle size (approximately 1 *μ*m) compared to the particle size of cement (approximately 12 *μ*m) further increases the density of the pore structure of metakaolin cement-based composites. 

### 3.5. Influence of Metakaolin on Microscopy Characteristics

**Figure 16:**
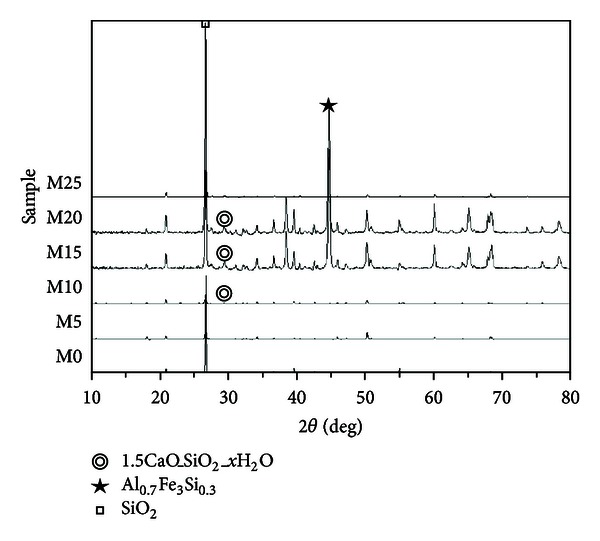
XRD results of paste specimens with metakaolin.

This study employed X-ray diffraction analysis to determine the products of hydration in cement-based materials.  Figure 16 illustrates the XRD results of cement-based paste specimens with replacement metakaolin at 56 days. It was found that the amounts of the primary hydration products, C-S-H (1.5CaO-SiO_2_-*x*H_2_O) and SiO_2_, present a significant form with the addition of metakaolin (M10, M15, and M20). In contrast, Al_0.7_Fe_3_Si_0.3_ content (peak value in Figure 16) increased significantly in the specimens containing 15% and 20% metakaolin. This is because metakaolin contains high quantities of Al_0.7_Fe_3_Si_0.3_, which verifies that nonhydrated particles could act as filler, changing large capillary pores into small, discontinuous capillary pores. The M15 and M20 specimens presented superior pozzolanic activity, which is consistent with the compressive strength test results, quantity of efflorescence, and SEM observations. In addition, the peak value for the M25 specimens dropped significantly, indicating an inferior hydrated reaction compared with the control specimens. This trend is similar to the results of compressive strength, in which the hydration reaction of specimens of samples with more than 20% metakaolin is lower than that of control specimens, due to the influence of reduced resistance to efflorescence.

## 4. Conclusion

Our results demonstrate that the replacement of cement using metakaolin at percentages of 5%, 10%, 15%, and 20% increases compressive strength with a peak increase in samples produced using 15% metakaolin. However, a distinct drop in compressive strength was observed in samples produced using 25% metakaolin. MATLAB image analysis and the curettage method both indicate that efflorescence is most pronounced when samples were cured in a low temperature environment, exceeding that produced under a normal environment or carbon dioxide environment. In the M5, M10, M15, and M20 samples, the area affected by efflorescence was lower than that of the control specimens; the M15 specimens were the least affected by efflorescence. In SEM micrographs, materials produced with metakaolin developed denser, smoother structures. XRD results indicate that the amount of main hydration products in cement-based materials with replacement metakaolin performed a significant form. Our results conclusively demonstrate that cement-based materials with 15% replacement metakaolin have superior performance with considerable potential for application in engineering.

## Figures and Tables

**Table 1:** Physical properties of metakaolin.

Item	Specific gravity	Color	Brightness (% ISO)	Surface area (m^2^/g)	D10 (*μ*m)	D50 (*μ*m)	D90 (*μ*m)	Bulk density (g/cm^3^)
Condition	2.60	White	79–82	15	<2.0	<4.5	<25	0.03~0.04

**Table 2:** Chemical properties of metakaolin.

Item	SiO_2_	Al_2_O_3_	Fe_2_O_3_	Tio_2_	SO_4_	P_2_O_5_	CaO	MgO	Na_2_O	K_2_O	L.O.I
Mass % as oxide	52–55	41–44	<1.9	<3.0	<0.05	<0.2	<0.2	<0.1	<0.05	<0.75	<0.5

**Table 3:** Designed mix proportions.

Mix	Water (g)	Cement (g)	Metakaolin (g)	Sand (g)
M0	417.47	834.94	0	834.94
M5	417.47	793.19	41.75	834.94
M10	417.47	751.45	83.49	834.94
M15	417.47	709.69	125.25	834.94
M20	417.47	667.96	166.98	834.94
M25	417.47	814.19	208.75	834.94

**Table 4:** Compressive strength and percent of relative compressive strength.

Mix	Compressive strength (MPa)				Relative compressive strength (%)			
	1 day	3 day	7 day	28 day	1 day	3 day	7 day	28 day
M0	12.62	25.20	28.66	40.22	100	100	100	100
M5	14.31	28.46	32.96	51.34	113	113	115	128
M10	14.78	30.54	39.18	53.46	117	121	137	133
M15	15.03	37.16	44.36	55.72	119	147	155	139
M20	14.68	28.34	36.46	50.02	116	112	127	124
M25	11.76	20.88	26.82	35.96	93	83	94	89

**Table 5:** Proportion of white efflorescence over total area (unit: %).

Mix	Exposure environment and age in days					
	7 days (NE)	56 days (NE)	7 days (CDE)	56 days (CDE)	7 days (LTE)	56 days (LTE)
M0	34.14	47.85	31.85	34.09	44.60	57.04
M5	33.89	42.85	36.57	39.61	36.01	52.94
M10	15.87	34.42	21.90	31.59	20.53	41.38
M15	15.14	20.34	9.25	10.25	15.29	21.71
M20	33.29	35.22	15.88	25.04	28.65	32.27
M25	46.35	49.20	24.24	29.06	30.07	44.06

**Table 6:** Quantity of efflorescence using the curettage method under NE (unit: g).

	M0	M5	M10	M15	M20	M25
Weight before curettage	1352.8	1397.6	1384.8	1353.9	1410.6	1261.7
Weight after curettage	1352	1397	1384.2	1353.5	1410.1	1260.7
Weight loss	0.8	0.6	0.6	0.4	0.5	1

**Table 7:** Quantity of efflorescence using the curettage method under CDE (unit: g).

	M0	M5	M10	M15	M20	M25
Weight before curettage	1328.4	1400.2	1414.7	1378.5	1418.7	1261.8
Weight after curettage	1327.8	1400	1414.5	1378.4	1418.6	1261.4
Weight loss	0.6	0.2	0.2	0.1	0.1	0.4

**Table 8:** Quantity of efflorescence using the curettage method under LTE (unit: g).

	M0	M5	M10	M15	M20	M25
Weight before curettage	1315.3	1290.2	1346.9	1314.7	1325.2	1252.4
Weight after curettage	1314.1	1289.4	1346.3	1314.2	1324.5	1251.3
Weight loss	1.2	0.8	0.6	0.5	0.7	1.1
